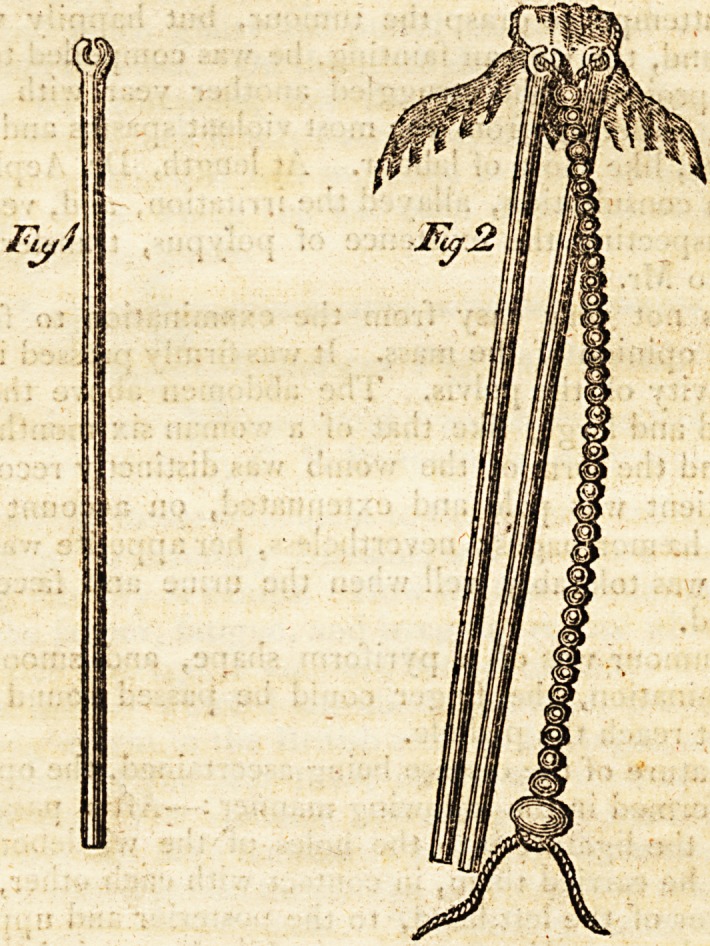# Description of an Improved Instrument for the Removal of Polypus Uteri, with a Case; from the German of Mr. Sauter

**Published:** 1814-10

**Authors:** 


THE
### Medical and Physical Journal.

4 OF VOL. XXXII.] OCTOBER, 1814. [NO. 188.

"For many fortunate discoveries in medicine, and for the detection of numerous errors, the world is indebted to the rapid circulation of Monthly Journals; and there never existed any work to which the faculty in EUROPE and AMERICA were under deeper obligations than to the Medical and Physical Journal of London, now forming a long, but an invaluable series."—RUSH.

*For the Medical and Physical Journal.*

### *Description of an improved Instrument for the Removal of Polypus Uteri, with a Case; from the German of Mr. Sauter; by* MR. WANT.

Fig 1

Fig 2

MR. SAUTER has published the description of this improved instrument for the removal of polypi of the womb, accompanied with four cases in which it has been used with success. One of these is of sufficient importance to be generally known, and will serve to show the manner of treating this distressing complaint.

A woman, 35 years of age, for these five years past has been subject to uterine hæmorrhages, and latterly to retention of urine and fæces, which threatened her life. A variety of physicians have been consulted, but the cause of the symptoms remained a long time in obscurity. As it became eventually necessary to evacuate the contents of the bladder by means of a catheter, it was discovered that the retention was occasioned by a solid and voluminous mass, which occupied the lower part of the pelvis. Oswald de Sommini, an ignorant and rash surgeon, who had attended the patient during her retention of urine, conceived, that by removing the solid and extraneous body, a radical cure might be obtained. He determined to perform the operation. He forcibly passed the hand into the womb, for the purpose of grasping and tearing away the mass, and exhorted the patient to commend her soul to God. He now made several attempts to grasp the tumour, but happily without effect; and, the woman fainting, he was compelled to abandon the project. She struggled another year with her afflictions, suffering from the most violent spasms and expulsive pains, like those of labour. At length, Dr. Aepli being called in consultation, allayed the irritation, and, very properly suspecting the existence of polypus, transferred the patient to Mr. S.

It was not very easy from the examination to form an accurate opinion of the mass. It was firmly pressed into the lesser cavity of the pelvis. The abdomen above the navel was hard and large, like that of a woman six months pregnant; and the form of the womb was distinctly recognised. The patient was pale and extenuated, on account of her frequent hæmorrhages; nevertheless, her appetite was good, and she was tolerably well when the urine and fæces were evacuated.

The tumour was of a pyriform shape, and smooth. In the examination, the finger could be passed round it, but could not reach the pedicle.

The nature of the disease being ascertained, the operation was performed in the following manner:—After passing the noose of the ligature into the holes of the whalebone conductors, he carried them, in contact with each other, on the fore-finger of the left hand, to the posterior and upper part of the polypus. He fixed one, while he carried the other round the tumour to its anterior part; then he did the same to that which was fixed, bringing it round on the opposite side, and connecting them together anteriorly. He pushed up the little balls on the ligatures, until the upper one reached the holes of the whalebone, and the ligature encircled circled the *base of the tumour*. Before he withdrew the conductors, he made a surgeon's knot at the lower extremity of the balls, by which means the neck of the tumour was firmly embraced. They were then withdrawn; and the whole operation was attended with little or no pain.

The part included in the ligature was ten inches from the orifice of the vagina; and the instrument, after being brought in contact on the anterior part of the tumour, was distinctly felt at the fundus of the uterus above the navel.

The two first days after the operation were good; but on the third, when the polypus tumefied, pains came on similar to those of labour, and the urine and fæces were completely suppressed. The ligature was again tightened as much as possible, which was repeated from day to day, and the flow of urine was assisted by lifting upwards the polypus. On the sixth day, phlyctenæ, occasioned by the mortification, evacuated much glairy and putrid matter, and the patient was much relieved. On the tenth day the separation of the tumour was complete, but the polypus remained in the pelvis, from which it was extracted with the forceps. Before the operation, it was supposed to have weighed at least a pound, since which she has been delivered of one child, and is again pregnant.

---

*For the Medical and Physical Journal.*

## Figures and Tables

**Fig 1 Fig 2 f1:**